# Supplementary material for: Limited Association between Schizophrenia Genetic Risk Factors and Transcriptomic Features
Source: Genes (Basel). 2021 Jul 12;12(7):1062. doi: 10.3390/genes12071062 (PMC8304922; doi:10.3390/genes12071062)
Supplement: Supplementary file 1 [file genes-12-01062-s001.zip › genes-1253582-supplementary.pdf]

Supplementary Materials

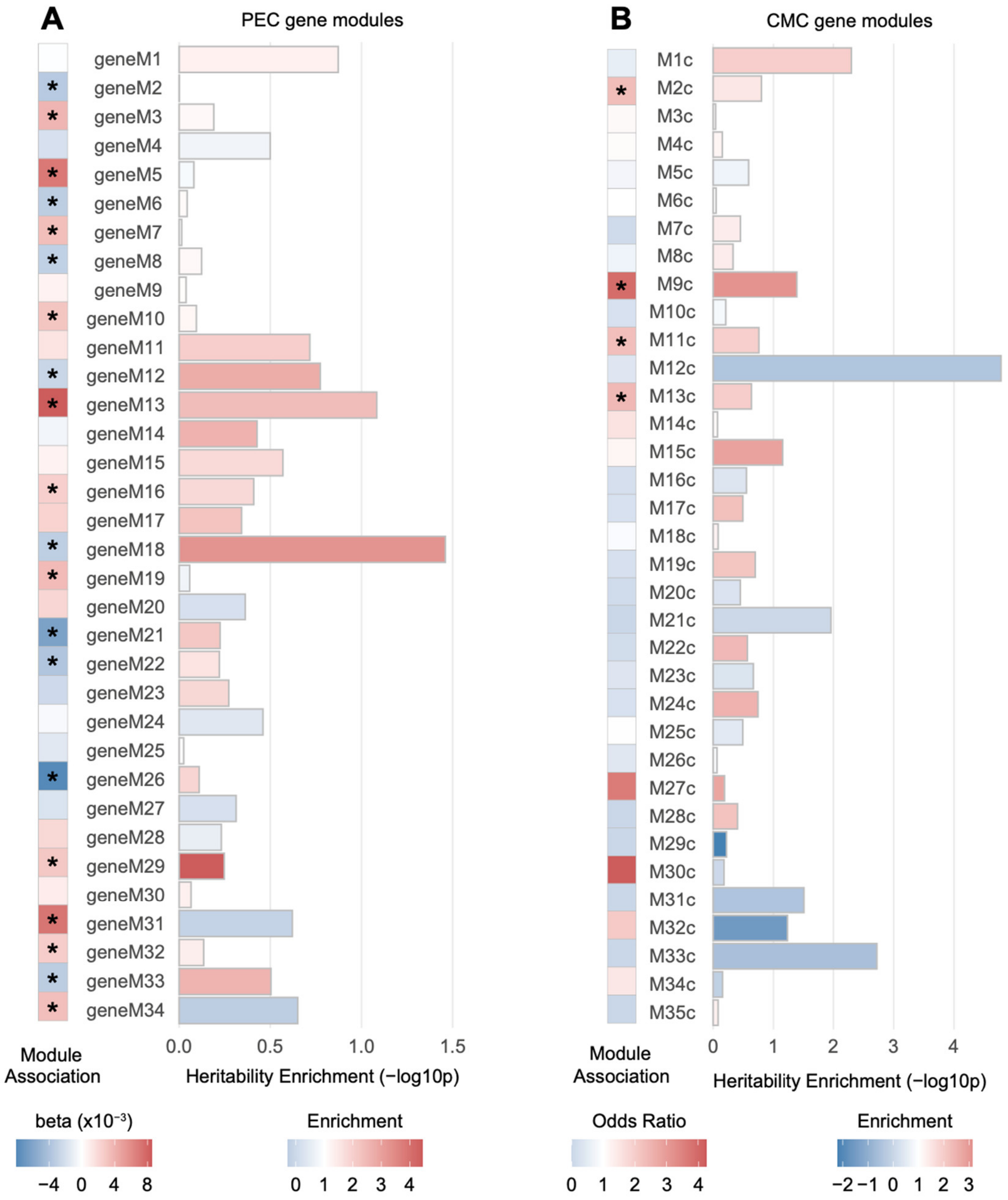

Figure S1. Schizophrenia heritability explained by gene co-expression modules associated with schizophrenia. (**A**) None of the PEC gene-level co-expression modules is enriched for schizophrenia heritability at a threshold of FDR<0.05 when genes in each module were mapped to SNPs on the basis of eQTL evidence. Co-expression modules marked with an asterisk (*) represents a PEC co-expression module significantly associated with schizophrenia. Module association denotes whether the eigengene of a given module is upregulated (red, $\beta > 0$) or downregulated (blue, $\beta < 0$) in schizophrenia. (**B**) No CMC gene-level co-expression module is enriched for schizophrenia heritability at a threshold of FDR<0.05 when genes in each module were mapped to SNPs on the basis of eQTL evidence. Co-expression modules marked with an asterisk (*) represents a CMC co-expression module enriched for DEGs in schizophrenia.

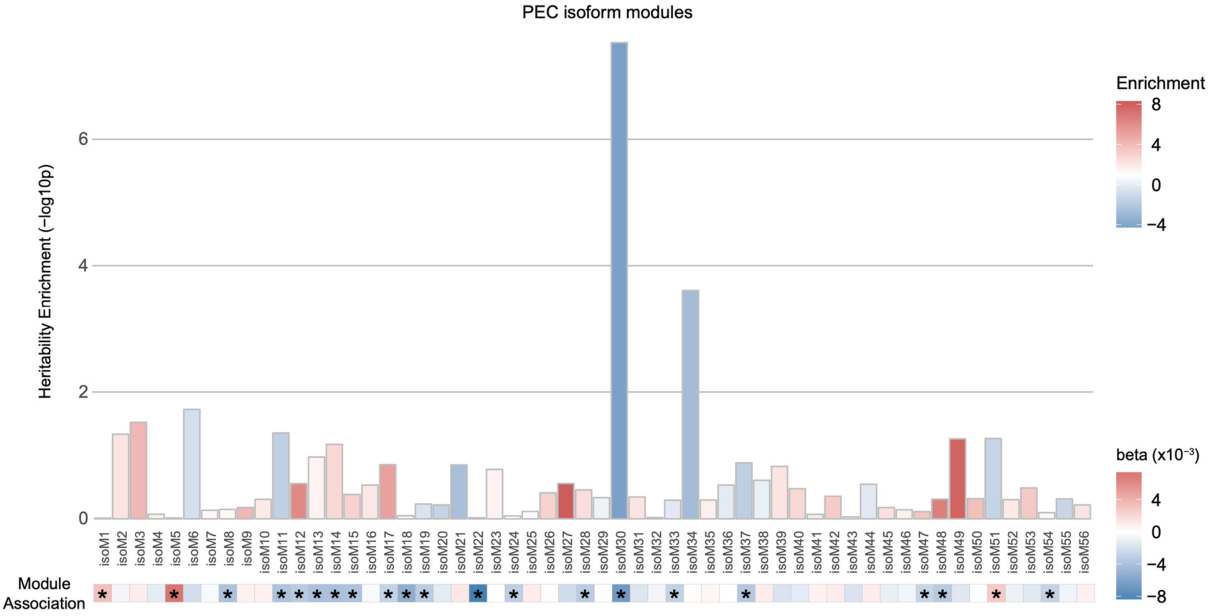

Figure S2. Heritability enrichment of isoform-level co-expression modules associated with schizophrenia. None of the PEC isoform-level co-expression modules is significantly enriched for schizophrenia heritability (FDR<0.05) when genes in the module were mapped to SNPs on the basis of isoQTL evidence. An asterisk (*) indicates isoform co-expression modules that are significantly associated with schizophrenia with eigengenes upregulated (red, $\beta > 0$) or downregulated (blue, $\beta < 0$) in schizophrenia postmortem brains.

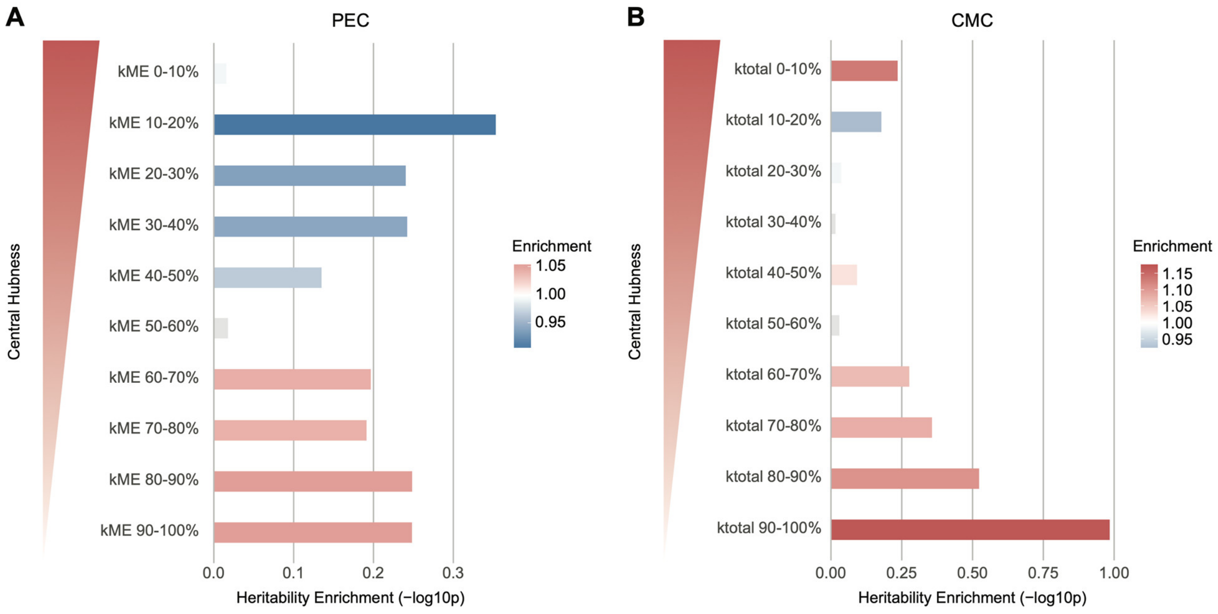

Figure S3. Relationship between central hubness of genes and heritability enrichment. None of the kME (**A**) or kTotal (**B**) groups is enriched for schizophrenia heritability when genes in each kME/kTotal group were mapped to SNPs on the basis of eQTL association.

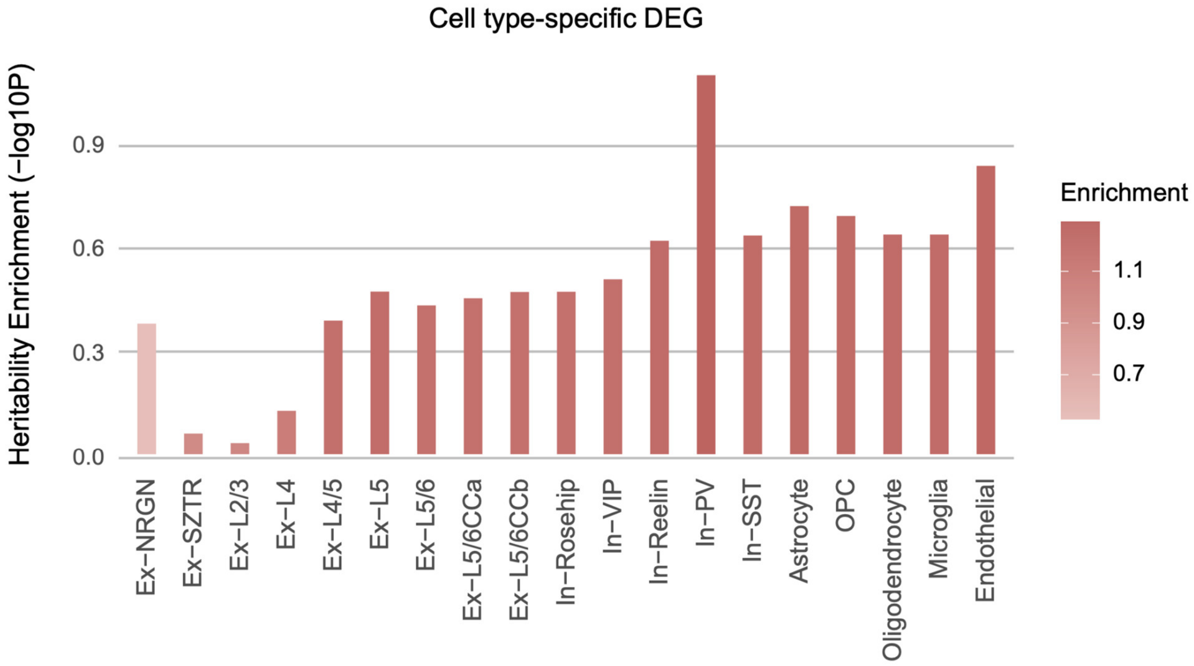

Figure S4. None of the cell types show significant heritability enrichment when SNPs were assigned to cell-type specific DEGs via eQTL association. Ex, excitatory neurons; In, inhibitory neurons; L, layer; NRGN, neurogranin; SZTR, schizophrenia transcriptional resilience; CC, cortico-cortical; VIP, vasoactive intestinal polypeptide; PV, parvalbumin; SST, somatostatin; OPC, oligodendrocyte progenitor cells.
